# Supplementary material for: Splice-Junction-Based Mapping of Alternative Isoforms in the Human Proteome
Source: Cell Rep. Author manuscript; Available in PMC 2020 Jan 15. (PMC6961840; doi:10.1016/j.celrep.2019.11.026)
Supplement: 3 [file NIHMS1546469-supplement-3.zip › DF2/PXD0010154/AdrenalGland-185-Q99715-SGPGTPLTGNAATEEER.pdf]

A

Predicted sequence disorder and sequence features of Q99715

Peptide: SGPGTPLTGNAATEEER Junction: sp|Q99715|COCA1_HUMAN|ENSG00000111799|SE2|40922|chr6|75165779|75175310|−2|r21|T1 TrNovel: TRUE

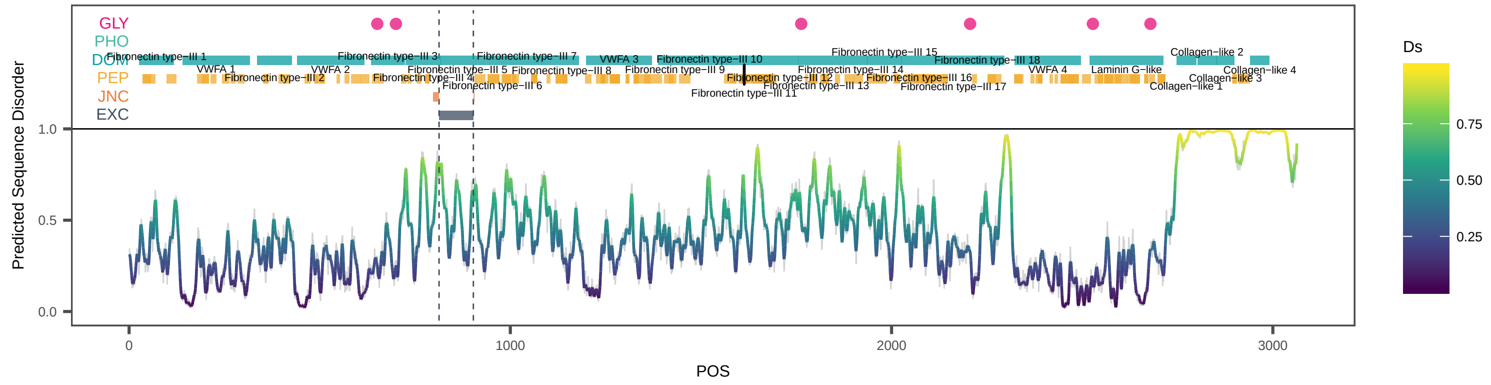

B

Distribution of sequence disorder in excised
vs. mapped and non−excised regions of protein

M−W P−value vs. mapped: 6.53e−13 vs. non−excised: 9.22e−07

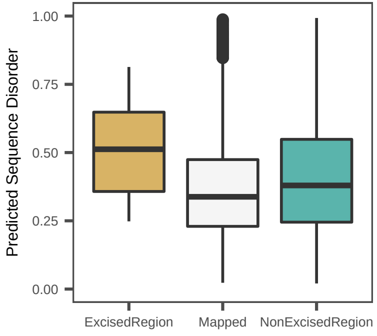

C
